# Associations of long-term exposure to ambient PM_1_ with hypertension and blood pressure in rural Chinese population: The Henan rural cohort study

**DOI:** 10.1016/j.envint.2019.04.037

**Published:** 2019-07

**Authors:** Na Li, Gongbo Chen, Feifei Liu, Shuyuan Mao, Yisi Liu, Yitan Hou, Yuanan Lu, Suyang Liu, Chongjian Wang, Hao Xiang, Yuming Guo, Shanshan Li

**Affiliations:** a Department of Global Health, School of Health Sciences, Wuhan University, 115# Donghu Road, Wuhan, China; b Department of Environmental and Occupational Health Sciences, University of Washington, 1959 NE Pacific Street, Seattle, USA; c Environmental Health Laboratory, Department of Public Health Sciences, University Hawaii at Manoa, 1960 East West Rd, Biomed Bldg, D105, Honolulu, USA; d Department of Epidemiology and Biostatistics, School of Public Health, Zhengzhou University, Zhengzhou, Henan, China; e Department of Epidemiology and Preventive Medicine, School of Public Health and Preventive Medicine, Monash University, Melbourne, Australia

**Keywords:** Air pollution, PM_1_, Hypertension, Blood pressure, Rural China

## Abstract

**Background:**

The epidemiological evidence on relationships between long-term exposure to particulate matter and hypertension and blood pressure has been inconclusive. Limited evidence was available for particulate matter with an aerodynamic diameter ≤ 1 μm (PM_1_) in rural areas of developing countries.

**Objective:**

This study aimed to investigate the associations between long-term exposure to PM_1_ and hypertension and blood pressure among rural Chinese population.

**Methods:**

This study included 39,259 participants who had completed the baseline survey from Henan Rural Cohort. Participants' exposure to PM_1_ was assessed by a satellite-based spatiotemporal model. The binary logistic regression model was used to examine the association between long-term PM_1_ exposure and hypertension, and multivariable linear regression model was used to investigate the associations between long-term PM_1_ exposure and systolic blood pressure (SBP), diastolic blood pressure (DBP), mean arterial pressure (MAP) and pulse pressure (PP). Moreover, we examined potential effect modifications by demographic, lifestyle and diet factors.

**Results:**

The mean concentration of PM_1_ for all participants during the 3-year before baseline survey was 59.98 μg/m^3^. Each 1 μg/m^3^ increase in PM_1_ concentration was significantly associated with an increase of 4.3% [Odds ratio(OR) = 1.043, 95% confidence interval(CI): 1.033, 1.053] in odds for hypertension, an increase of 0.401 mm Hg (95% CI, 0.335, 0.467), 0.328 mm Hg (95% CI, 0.288, 0.369), 0.353 mm Hg (95% CI, 0.307, 0.399) and 0.073 mm Hg (95% CI, 0.030, 0.116) in SBP, DBP, MAP and PP, respectively. Further stratified analyses showed that the effect of PM_1_ on hypertension and blood pressure could be modified by sex, lifestyle and diet.

**Conclusions:**

This study suggests that long-term exposure to ambient PM_1_ increases the risk of hypertension and is associated with elevations in blood pressure in rural Chinese adults, especially in male and those with unhealthy habits.

## Highlights

• Rural cohort participants were exposed to high level of PM_1_ during the study period.
• PM_1_ was related to increased risk of hypertension and elevated blood pressure.
• Males and those with unhealthy habits were more susceptible to the adverse effect.

## 1 Introduction

Hypertension is a well-established risk factor for cardiovascular disease that contributed to 17.8 million deaths worldwide in 2017 (Brook et al., 2010; GBD, 2018). Among all risk factors, high systolic blood pressure (SBP) has been identified as the largest contributor of all-cause deaths worldwide during the period from 1990 through 2017, accounting for 10.4 million deaths and 218 million disability-adjusted life lost years (DALYs) in 2017 (GBD, 2018). During the past four decades, the number of people with high blood pressure has been rising worldwide, especially in low-income and middle-income countries (Zhou et al., 2017). In China, blood pressure control is a national public health priority (Chen, 2009), as nearly half of adults aging 35–75 years are suffering from hypertension and the prevalence has been rising (Lu et al., 2017). In addition to genetic factors, changes in lifestyle and diet habits, deteriorating environmental condition has been recognized as a risk factor for increased blood pressure as well (Argacha et al., 2018).

In recent years, mounting evidence contributed to a better understanding of the associations between exposure to ambient air pollutants and elevated BP and hypertension. A comprehensive meta-analysis reviewed the existing evidence and reported globally significant associations of long-term exposure to fine particulate matter (particle matter with an aerodynamic diameter ≤ 2.5 μm, PM_2.5_) with hypertension, and of particulate matter with an aerodynamic diameter ≤ 10 μm (PM_10_), PM_2.5_, and nitrogen dioxide (NO_2_) with increased diastolic blood pressure (DBP) (Yang et al., 2018a). However, high heterogeneity was found for this meta-analysis and the existing literature on long-term exposure to air pollution and blood pressure mainly focused on pollutants such as PM_10_, PM_2.5_ and NO_2_. Few researches have evaluated the cardiovascular effects of long-term exposure to PM_1_ that is the important component of PM_2.5_ and may have more extensive toxic effects than PM_2.5_ (Chen et al., 2017). In addition to SBP and diastolic blood pressure (DBP), increased mean arterial pressure (MAP) and pulse pressure (PP), the steady component and pulsatile component of blood pressure respectively, have been reported to be related to higher cardiovascular risk as well (Darne et al., 1989; Madhavan et al., 1994). However, compared with SBP and DBP, far less evidence has been available for air pollution and MAP/PP. Moreover, the majority of previous studies were conducted in urban areas. They paid little attention to rural areas in developing countries, where hypertension is becoming much more prevalent and air pollution is worsening.

In this context, to fill in the gap and add to the evidence for adverse effect of long-term exposure to PM_1_, we investigated the associations between long-term exposure to PM_1_ and hypertension and four blood pressure component measurements in rural Chinese population. We also examined several potential effect modifications by demographic, lifestyle and diet factors.

## 2 Methods

### 2.1 Study population

We studied participants from The Henan Rural Cohort Study (Registration number: ChiCTR-OOC-15006699), which was established in five rural areas (Tongxu county of Kaifeng city, Yima county of Sanmenxia city, Suiping county of Zhumadian city, Xinxiang county of Xinxiang city and Yuzhou county of Xuchang city) of Henan Province, China, in July 2015. The detailed descriptions of cohort design and study population were previously published elsewhere (Liu et al., 2019; Tian et al., 2018a; Tian et al., 2018b). Briefly, the cohort recruited participants from the general population using multistage stratified cluster sampling method. The target population were 18- to 79-year-old permanent residents without severe physical or mental disease. In the first stage, five rural counties in different geographical regions (south, central, north, east, and west) of Henan Province were selected by simple cluster sampling. In the second stage, in view of the compliance of the residents, population stability and local medical conditions, one to three rural communities (referred to as a “township”) in each county were selected by the local Centre for Disease Control and Prevention. In the final stage, the eligible candidates who signed informed consent in each administrative unit (rural village) of the selected township were included in the study sample. Overall, a total of 41,893 invitations were sent out to those who met the inclusion criteria and 39,259 participants responded and completed baseline survey, with a response rate of 93.7% (Liu et al., 2018). In this study, 52 participants were excluded due to missing data about blood pressure measurement, hypertension status, and other key covariates. Therefore, this analysis included 39,207 (99.9%) of the recruited participants completing the baseline survey. Data on demographic characteristics, socioeconomic characteristics, health behaviors, physician-diagnosed diseases, medication history, and family history of diseases were collected via face-to-face interviews by well-trained local investigators.

The study complied with the 1975 Declaration of Helsinki and was approved by the ethics committee of Zhengzhou University. Written informed consent was obtained from each participant at their enrollment.

### 2.2 Air pollution exposure assessment

Daily PM_1_ concentrations were predicted at a 0.1° × 0.1° spatial resolution, using satellite remote sensing, meteorology, and land use information. The detailed description of the prediction has been previously published (Chen et al., 2018a; Chen et al., 2018b). In brief, we combined two types of Moderate Resolution Imaging Spectroradiometer (MODIS) Collection 6 aerosol optical depth (AOD) data, Dark Target (DT) and Deep Blue (DB). A random forests model based on machine learning algorithms was employed to model ground-monitored PM_1_ data with AOD data and other spatial and temporal predictors (e.g., urban cover, forest cover and calendar month). A 10-fold cross-validation was performed to assess the predictive ability. The results of 10-fold cross-validation showed that R^2^ and Root Mean Squared Error (RMSE) for daily prediction was 64% and 17 μg/m^3^. For annual prediction, R^2^ and RMSE was 82% and 9 μg/m^3^, respectively. We assigned PM_1_ concentration estimates for each participant according to the geocoded address of natural village through AutoNavi Map (https://lbs.amap.com/api/webservice/guide/api/georegeo). This is a Chinese web mapping, navigation and location-based services provider and has provided mapping data to Google since 2006. Then we calculated the average during the three-year before the baseline survey for each participant as long-term exposure concentration of PM_1_.

### 2.3 Outcome assessment

Blood pressure was measured using an electronic sphygmomanometer (HEM-770A Fuzzy, Omron, Kyoto, Japan) in the sitting position for three times according to the American Heart Association's standardized protocol (Perloff et al., 1993). Participants were advised not to smoke, drink alcohol, coffee, or tea, and to abstain from exercising for at least 30 min before measurement. Additionally, they were not allowed to talk during the measurement. The average value of three measurements was used as the blood pressure measurement for this study. Mean arterial pressure was calculated as DBP + 1/3 (SBP − DBP); pulse pressure was calculated as the difference between SBP and DBP (Darne et al., 1989). According to the 2010 Chinese guidelines for the management of hypertension (Liu, 2011), hypertension in our analysis was defined as having a measured SBP ≥140 mm Hg or DBP ≥ 90 mm Hg, or having a self-report of either physician-diagnosed hypertension or anti-hypertension treatment.

### 2.4 Covariates

We controlled for potential confounders based on the previous literature on air pollution and blood pressure. Demographic covariates included age and sex. Socioeconomic covariates included education level (“low”, “medium” or “high”), marital status (“married/cohabitating” or “widowed/single/divorced/separation”), average monthly income (“≤500 RMB”, “500–1000 RMB” or “≥1000 RMB”). Health behavior covariates included smoking status (“never smoking” or “ever smoking”), alcohol drinking status (“never drinking” or “ever drinking”), high fat diet (“yes” or “no”), more vegetables and fruits intake (“yes” or “no”), physical activity (“low level”, “moderate level” or “high level”). Health status covariates included body mass index (BMI), family history of hypertension, and type 2 diabetes. Regarding education level, participants with no schooling or participants who attained up to primary school were considered as low education level, while those who graduated from junior school were considered as medium and those who graduated from senior high school or above were considered as high education level. For smoking status, the responses of “former smoking” and “current smoking” were merged into the variable of “ever smoking” in order to further analysis and make comparison with other studies. This is also the case for drinking status. According to Chinese dietary guidelines (SS Wang et al. 2016), “More vegetables and fruits intake” was defined as average intake of 500 g or more vegetables and fruits per day. High fat diet was defined as consumptions of 75 g or more meat from livestock and poultry per day. Physical activity was divided into three levels according to the international physical activity questionnaire (IPAQ) (Lee et al., 2011): low, moderate, and high. Diabetes was defined as having a fasting plasma glucose (FPG) ≥ 7.0 mmol/L, and/or diagnosed as diabetes by a physician (American Diabetes, 2013).

### 2.5 Statistical analysis

Descriptive analyses were conducted for all variables. Continuous variables were described as mean ± standard deviations (SD) and categorical variables were expressed as counts and percentage. Differences in the distribution of baseline characteristics between groups were tested using Mann-Whitney *U* test for continuous variables and chi-square test for categorical variables. We employed the logistic regression model to examine the association between long-term PM_1_ exposure and hypertension as a dichotomous outcome. And multivariate linear regression models were employed to investigate associations between PM_1_ exposure and blood pressure component as a continuous measure. The effect estimates were presented as odds ratios (ORs) for hypertension and changes in mm Hg for blood pressure measures per 1 μg/m^3^ increment in PM_1_ concentration, with corresponding 95% confidence intervals (CIs).

We also examined whether the PM_1_-hypertension and PM_1_-BP associations were potentially modified by sex, age, smoking status, drinking status, high-fat diet, more vegetables and fruits intake and physical activity. We did subgroup analyses by each potential modifier, and a cross-product term was added into separate models to assess the significance of interaction terms (Liu et al., 2017). The sensitivity analysis was performed to examine the robustness of results. We did sensitivity analyses using average concentrations of PM_1_ for 1, 2, 4, and 5 years before the survey to evaluate the long-term effects of PM_1_ exposure, and by adjusting for survey site as a covariate.

All the statistical analyses were completed using R 3.5.0 (R Foundation for Statistical Computing, 2004 (ISBN 3-900051-00-3), Vienna, Austria).

## 3 Results

**Fig. 1:**
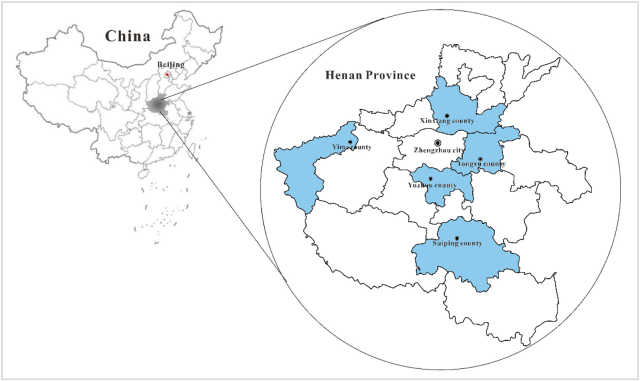
The locations of five survey sites in the Henan Rural Cohort Study. Note: The map of China was downloaded from the national administration of surveying, mapping and geoinformation (Available at: http://bzdt.nasg.gov.cn/index.jsp. Accessed on 27 January, 2018), and the serial number was 8012790168. The map of Henan Province was generated by Map institution of Henan Province.

Fig. 1 shows the locations of five survey sites in the Henan Rural Cohort Study. The basic characteristics of all participants are shown in Table 1. Additionally, we also presented basic characteristic of participants by five survey sites in Table S1.The mean age of all participants was 55.6 (SD:12.19) years, and the majority were female (60.1%). In total, 12,823 hypertension cases were identified with the prevalence of 32.7%. Among the hypertension cases, 7879 (61.4%) were self-reported and 4955 (38.6%) were diagnosed as hypertension in the survey, 6319 (49.2%) had taken antihypertensive medication during the past two weeks. Participants with hypertension tended to be older (60.39 versus 53.27, *P* < 0.001), had a higher level of BMI (26.02 versus 24.21, *P* < 0.001) than normotensive participants. Also, they were more likely to have family history of hypertension (27.5% versus 15.4% *P* < 0.001) and diabetes (14.8% versus 6.9%, *P* < 0.001) than normotensive participants. The 3-year average concentration of PM_1_ for overall cohort was 55.99 μg/m^3^ (SD:2.06), and the hypertensive participants had similar PM_1_ exposure levels with normotensive participants (57.72 μg/m^3^ and 57.31 μg/m^3^, respectively).

**Table 1:** Basic characteristics of study participants.

Characteristics^a^	Non-hypertension (n = 26,384)	Hypertension (n = 12,823)	*P* value
Age, years	53.27 ± 12.43	60.39 ± 10.09	<0.001
PM_1_, μg/m^3^	57.31 ± 2.67	57.72 ± 2.62	<0.001
Body mass index, kg/m^3^	24.25 ± 3.36	26.04 ± 3.67	<0.001
Systolic blood pressure, mm Hg	115.82 ± 11.91	146.81 ± 16.86	<0.001
Diastolic blood pressure, mm Hg	72.58 ± 8.14	88.22 ± 10.67	<0.001
Mean arterial pressure, mm Hg	87.00 ± 8.64	107.75 ± 11.12	<0.001
Pulse pressure, mm Hg	43.23 ± 8.68	58.59 ± 14.50	<0.001
Sex			0.170
Male	10,348 (39.2)	5127 (39.9)	
Female	16,036 (60.8)	7709 (60.1)	
Educational level			<0.001
Low	10,722 (40.6)	6834 (53.2)	
Medium	11,210 (42.5)	4418 (34.4)	
High	4452(16.9)	1584 (12.3)	
Marital status			<0.001
Married or cohabiting	24,023 (91.1)	11,185 (87.1)	
Widowed/single/divorced/separation	2361 (8.9)	1651 (12.9)	
Income			<0.001
≤500 RMB	8924 (33.8)	5078 (39.6)	
500–1000 RMB	8667 (32.8)	4226 (32.9)	
≥1000 RMB	8793 (33.3)	3532 (27.5)	
Smoking			0.131
Never	19,145 (72.6)	9407 (73.2)	
Ever	7239 (27.4)	3429 (26.7)	
Drinking			0.010
Never	20,496 (77.7)	9823 (76.5)	
Ever	5888 (22.3)	3013 (23.5)	
Physical activity			<0.001
Low	7856 (29.8)	4841 (37.7)	
Moderate	10,409 (39.5)	4386 (34.2)	
High	8119 (30.8)	3609 (28.1)	
High fat diet	5461 (20.7)	2012 (15.7)	<0.001
More vegetables and fruits intake	11,748 (44.5)	4625 (36.0)	<0.001
Family history of hypertension	4055 (15.4)	3535 (27.5)	<0.001
Type 2 diabetes	1808 (6.9)	1896 (14.8)	<0.001

a Note: Data are the mean ± standard deviation for continuous variables and number (percentage) for categorical variables.

For all participants, the OR of hypertension associated with 1 μg/m^3^ increment in PM_1_ concentration was 1.059 (95%CI: 1.051, 1.068) in crude model and 1.043 (95%CI: 1.033, 1.053) after fully adjusting for potential confounders (Adjusted model 2). Based on the final adjusted model (Adjusted model 2), each 1 μg/m^3^ increase in PM_1_ concentration was associated with an elevation of 0.401 (95% CI: 0.335, 0.467) mm Hg in SBP, 0.328 (95% CI: 0.288, 0.369) mm Hg in DBP, 0.353 (95% CI: 0.307, 0.399) mm Hg in MAP and 0.073 (95% CI: 0.030, 0.116) mm Hg in PP (Table 2).

**Table 2:** Odds ratios of hypertension and changes in blood pressure (mm Hg) associated with per 1 μg/m^3^ increment in long-term exposure to PM_1_.

Effect estimates	Crude model^a^	Adjusted model 1^b^	Adjusted model 2^c^
OR (95%CI)	1.059 (1.051, 1.068)	1.075 (1.066, 1.084)	1.043 (1.033, 1.053)
Changes in mm Hg (95% CI)			
SBP	0.633 (0.559, 0.707)	0.749 (0.680, 0.818)	0.401 (0.335, 0.467)
DBP	0.539 (0.496, 0.582)	0.553 (0.510, 0.596)	0.328 (0.288, 0.369)
MAP	0.570 (0.520, 0.621)	0.618 (0.569, 0.667)	0.353 (0.307, 0.399)
PP	0.094 (0.045, 0.143)	0.196 (0.154, 0.238)	0.073 (0.030, 0.116)

Abbreviations: PM_1_, particle matter with aerodynamic diameter ≤ 1.0 μm; OR, odds ratio; CI, confidence interval; SBP, systolic blood pressure; DBP, diastolic blood pressure; MAP, mean arterial pressure; PP, pulse pressure.

a Crude Model: no adjustment.

b Adjusted Model 1: adjusted for age, sex, marital status, education level, income.

c Adjusted Model 2: also adjusted for smoking, alcohol drinking, vegetables and fruits intake, high fat diet, physical activity, family history of hypertension, body mass index, type 2 diabetes and hypertension medicine use (not in logistic regression).

**Fig. 2:**
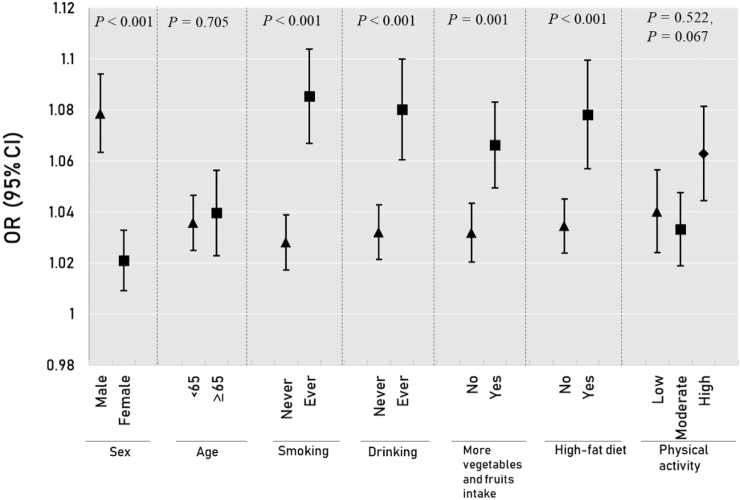
Odds ratio (95% confidence intervals) of hypertension in association with an increment of 1 μg/m^3^ in PM_1_ concentration, stratified by potential modifiers. Notes: Adjusted for sex, age, marital status, education level, income, smoking, alcohol drinking, physical activity, vegetables and fruits intake, high fat diet, family history of hypertension, body mass index, type 2 diabetes; *P* denotes the *p*-value for interaction term.

**Fig. 3:**
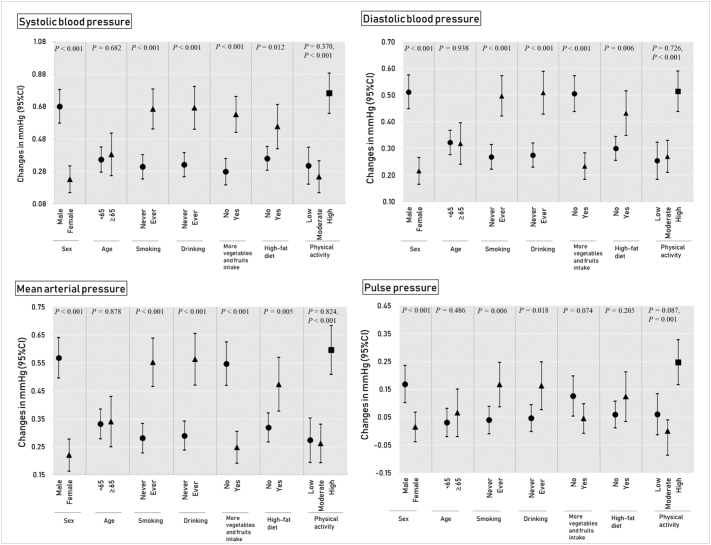
Changes in four blood pressure components (mm Hg) associated with an increment of 1 μg/m^3^ in long-term PM_1_ concentration, stratified by potential modifiers. Notes: Adjusted for sex, age, marital status, education level, income, smoking, alcohol drinking, physical activity, vegetables and fruits intake, high fat diet, family history of hypertension, body mass index, type 2 diabetes, hypertension medicine use; *P* denotes the *p*-value for interaction term.

The results of stratified analysis for hypertension are shown in Fig. 2 and Table S2. The ORs of hypertension associated with each 1 μg/m^3^ increase in PM_1_ were significantly higher among males, ever-smokers, ever drinkers, those who had more vegetables and fruits intake and those with a high-fat diet (Fig. 2 and Table S2). Fig. 3 and Table S3 presents the results of stratified analysis for blood pressure measurements. The associations between PM_1_ and four blood pressure component measurements were modified by sex, smoking status, drinking status and physical activity (*P* for interaction <0.05). We observed stronger positive associations in males, ever-smokers, ever-drinkers and participants with high level of physical activity. Except for PP, the effect modification by more vegetables and fruits intake and high-fat diet were also observed for SBP, DBP and MAP (Fig. 3 and Table S3).

The results of sensitivity analysis are presented in Table S4. The effect estimates of associations between PM_1_ exposure and hypertension did not change substantially after using different average concentration of PM_1_ but adjusting for the survey site. The results of PM_1_ and blood pressure measurements remained robust in all types of sensitivity analysis (comparing Table S4 with Table 2).

## 4 Discussion

This study suggested that long-term exposure to PM_1_ was significantly associated with increased prevalence of hypertension and elevated blood pressure in rural Chinese adults. To the best of our knowledge, this is the first study to investigate associations between exposure to PM_1_ and prevalence of hypertension and blood pressure in rural China. We used baseline data from a large cohort study with strict quality control and standardized assessment of outcome and confounders. Our findings can add further to the growing body of evidence that particulate matter can be an important risk factor for hypertension and perturbations in blood pressure. Considering the large population of hypertensive patients and the severity of air pollution, our findings are of remarkable significance for public health.

There is an increasing number of studies evaluating long-term effects of exposure to particulate air pollutants on hypertension and blood pressure in China. For example, Liu et al. reported that an inter quartile range increase (IQR, 41.7 μg/m^3^) in PM_2.5_ concentration was associated with an OR of 1.11 (95% CI: 1.05, 1.17) for hypertension and an increment of 0.60 mm Hg (95% CI: 0.05, 1.15) in SBP (Liu et al., 2017). A study conducted among older Chinese adults showed that each 10 μg/m^3^ increase in PM_2.5_ concentration was related to an OR of 1.14 (95% CI:1.07, 1.22) for hypertension, an increment of 1.30 mm Hg (95% CI: 0.04, 3.56) in SBP and an increment of 1.04 mm Hg (95% CI: 0.31, 1.78) in DBP (Lin et al., 2017b). A recent meta-analysis pooled 20 studies globally and reported a significant association between long-term exposure to PM_2.5_ and hypertension (OR = 1.05; 95% CI: 1.01, 1.09) (Yang et al., 2018a). However, far less information is available regarding the effect of long-term exposure to PM_1_ on hypertension and blood pressure. In fact, some current researches have indicated that the size and surface area were critical characteristics of particulate matter that determine their potential to elicit biological effects (Nel, 2005; Valavanidis et al., 2008). Smaller particles, such as PM_1_ and ultrafine particles, have higher surface-to-volumes ratios and can penetrate deeper into the airways of the respiratory tract. Also, they are more likely to persist in the lung parenchyma. Thus, smaller particles can elicit stronger biological effects like oxidative damage and inflammatory injury (Valavanidis et al., 2008). Additionally, it has been reported that most of the toxic metals tended to accumulate in the smaller particles (PM_2.5_ or less) and therefore PM_1_ may contain more toxins from anthropogenic emissions, which may even lead to gene damage (Izhar et al., 2016; Ravindra et al., 2008). However, neither China nor other countries has set air quality standards and guidelines for PM_1_ because of insufficiency of scientific evidence on this pollutant and unavailability of technologies for PM_1_ measurements (Wang and Hao, 2012). Along with other studies on health effects of PM_1_ (Chen et al., 2017; Lin et al., 2016; Yang et al., 2018b), the findings from this study can provide evidence for policy makers when promulgating standards and guidelines for the control of PM_1_ pollution.

Our study found that long-term exposure to ambient PM_1_ was positively associated with all four blood pressure component measurements, while previous studies showed inconsistent results regarding air pollution and blood pressure. A recent study conducted among reproductive-age adults in China reported that long-term exposure to PM_2.5_ was in association with increment of both SBP and DBP (Xie et al., 2018). Similar results have also been reported in a cross-sectional study in Taiwanese adults (Zhang et al., 2018). However, some studies showed different results. Liu et al. observed a significant association of PM_2.5_ and SBP, whereas a null association for DBP, in a nation-wide study of Chinese adults aging over 35 years old (Liu et al., 2017). The findings from a cohort of older Americans suggested that long-term exposure to PM_2.5_ was correlated with SBP but not with DBP (Honda et al., 2018). And yet, Chen et al. observed isolated elevations in DBP, but not SBP, with 1-year exposure to air pollution among elderly residents of Taipei City (Chen et al., 2015). These mixed results may be attributed to differences in characteristics of study population, sources or compositions of air pollutants, and measurements of outcomes, as well as different statistical methods (Cai et al., 2016).

Each blood pressure component measurement quantifies an aspect of cardiovascular function, and elevations in any component may lead to increased risk for cardiovascular outcomes. (Darne et al., 1989; Franklin et al., 2001; Honda et al., 2018; Kannel et al., 1981). Our results showed significant associations of long-term PM_1_ exposure with all four blood pressure component measurements, which could be relevant for public health initiatives. According to a series of studies, diastolic blood pressure was found to be the strongest predictor of coronary heart disease (CHD) development (HR per 10 mm Hg increment, 1.34; 95% CI, 1.18–1.51) in individuals younger than 50 years old (Franklin et al., 2001), whereas systolic blood pressure elevations were found to be the greatest risk for incident congestive heart failure (HR per 10 mm Hg increment, 1.56; 95% CI,1.37, 1.77) among adults aging 50–79 years (Haider et al., 2003). Furthermore, the existing research showed that a “small” reduction of 2 mm Hg in the mean of SBP has been estimated to reduce 25% stroke events in the population (Girerd and Giral, 2004). The possible biological mechanism by which particulate matter raised blood pressure includes the elicitation of oxidative stress, systemic inflammation, endothelial dysfunction and DNA methylation (Brook et al., 2010; Pope and Dockery, 2006; C Wang et al. 2016). The activation of pulmonary reflexes induced by inhalation of particulate matter may cause autonomic nervous system imbalance and arterial remodeling (Brook et al., 2009). Hypertrophic remodeling of resistance vessels may lead to medial thickness, resulting in BP elevations (Valavanidis et al., 2008).

Health effects of particulate air pollution can be modified by many factors. It is of great significance to identify subgroups who are potentially susceptible to the adverse effects of particulate air pollution. Our findings showed a significantly larger effect of PM_1_ exposure on hypertension and blood pressure in males than females. As prior evidence proposed, this sexual discrepancy could be attributed to some biological characteristics such as hormones, function of smooth muscle and vascular, airway and lung size (citation). Also, this sex-based difference in effect estimates can be related to some behavioral characteristics (e.g. outdoor activity, smoking and drinking). Men may accumulate greater PM_1_ exposures through long periods of physical activity (Clougherty, 2010). In addition, we observed larger ORs of hypertension and higher elevations in blood pressure for participants who were ever smokers and ever drinkers compared with never-smokers and never-drinkers. Existing evidence has indicated that both smoking and drinking can promote inflammatory response and oxidative stress (Bazzano et al., 2003; O'Keefe and Gheewala, 2008; Piano, 2017), which was identified as main biological mechanism of health damage induced by air pollution. Also, drinking can promote mitochondrial dysfunction, programmed cell death and anatomical damage to the cardiovascular system, especially to heart itself (Piano, 2017).

Previous epidemiological studies found that higher consumption of fruits and vegetables may mitigate the cardiovascular effects of particulate matter (Lin et al., 2017a; Lin et al., 2017b), which agreed with hypothesized mechanisms that air pollution induced health damage mainly through oxidative stress. More intake of fruits and vegetables improved the status of anti-oxidants and anti-inflammatory micronutrient status such as vitamin C, E, carotenoids, B group vitamins and flavonoids, which is in relation to an effect reduction in oxidative stress, inflammation and other adverse reactions induced by ambient air pollutants (Bowler and Crapo, 2002; Lin et al., 2017b; Romieu et al., 1998). However, higher ORs of hypertension and larger elevations in blood pressure were observed among those who had more intake of fruits and vegetables (≥500 g per day) in this study, which should be interpreted cautiously. Such inconsistence might be due to exposure misclassification about intake of vegetables and fruits. Firstly, we considered vegetables consumption and fruits consumption together whereas it was categorized as vegetables intake and fruits intake separately in other studies. Secondly, the information on type of vegetables and fruits, cooking method and frequency of intake are insufficient, and theses information is critical in examining the effect of fruits and vegetables intake on air pollution exposure. Because Chinese accustomed to eating cooked vegetables and there may be destruction and reduction in antioxidant components during the process of cooking. (Lin et al., 2017b).

In addition, those who had a high-fat diet were found to have greater risks of hypertension and larger increases in SBP, DBP and MAP. This could be explained that high-fat diet played an important role in the development of obesity (Schrauwen and Westerterp, 2000), which may substantially amplify the associations between air pollution and increased blood pressure, even the susceptibility to the adverse health effects of air pollutants (Bray and Popkin, 1998; Zhao et al., 2013). A significantly larger PM_1_-induced increase in blood pressure was observed among participants who had high-level physical activity compared with those who had low-level physical activity. Similar results were also reported in studies on the associations between PM_2.5_ exposure and other cardiovascular outcomes (Lin et al., 2017a). The possible mechanisms might be that people may have a higher level of exposure to air pollutants during the outdoor physical activities because of the increased breathing rates and intensity (Weichenthal et al., 2014). Thus physically active people had an increased inhalation and deposition of air pollutants in the body, leading to the amplification of harmful effects of air pollution (Strak et al., 2010).

Several limitations in our study should also be noted. First, this study used the baseline survey data of the cohort and the onset date of hypertension for each case was unknown. The cross-sectional design of study restricted us to a non-causal relationship between PM_1_ exposure and hypertension and blood pressure. Second, the individual exposure to ambient PM_1_ showed very small differences among the overall cohort, for the reason that all of five study sites were seriously polluted areas. Third, demographic information and lifestyle characteristics were collected using a questionnaire, thus recall bias may not be avoided. Forth, traffic noise was another possible uncontrolled confounder in this study, which has been suggested to associate with elevated blood pressure and other cardiovascular events (Chang et al., 2007; Dzhambov and Dimitrova, 2018; Sears et al., 2018). However, considering the cohort study was conducted in rural areas and roads in rural areas are mostly village roads or country lanes, there is no heavy traffic and severe noise in rural areas compared with urban areas. Thus we suspected that rural people may not be impacted by the traffic noise and this may not have considerable impact on our results. In addition, road condition data in rural areas are relatively scarce in China. Finally, there potentially exists larger spatial error of misclassification when geocoding rural address compared with geocoding address in urban environments.

## 5 Conclusion

This study demonstrated that long-term exposure to PM_1_ was significantly associated with increased risk of hypertension and elevated blood pressure measurements in rural Chinese adults. Males, ever-smokers, ever-drinkers, those with high-fat diet and high-level physical activity might be more susceptible to the adverse effect of long-term PM_1_ exposure. Our findings added further to evidence that PM_1_ exposure could be an important risk factor for hypertension and perturbations in blood pressure. In addition, these findings also suggests a necessity for including an index for the concentration of PM_1_ in Chinese air quality standards for the purpose of more effective mitigation of air pollution in China.

## Declarations of interest

The authors declare no conflicts of interest.

## Funding

The authors acknowledge the cooperation of all the participants and administrators in this study. This research was supported by the Foundation of National Key Research and Development Program of China (Grant NO: 2016YFC0900803) and the Natural Science Fund of Hubei Province (Granter number: 2018CFB634). Dr. Guo is supported by Career Development Fellowship APP1107107 from the Australian National Health and Medical Research Council (NHMRC). Dr. S. Li is supported by Early Career Fellowship APP1109193 from the Australian NHMRC.

## Appendix A Supplementary data

Supplementary tables Image 1
