# Supplementary material for: Associations of long-term exposure to ambient PM1 with hypertension and blood pressure in rural Chinese population: The Henan rural cohort study
Source: Environ Int. 2019 Jul;128:95–102. doi: 10.1016/j.envint.2019.04.037 (PMC7086153; doi:10.1016/j.envint.2019.04.037)
Supplement: Supplementary file 1 — Supplementary tables [file mmc1.docx]

**Supplementary**

**Table S1** Basic demographic and socio-economic characteristics of study participants by five survey sites

| Characteristics | **Yuzhou** | |  | **Zhumadian** | |  | **Kaifen** | |  | **Xinxiang** | |  | **Yima** | |  |
| --- | --- | --- | --- | --- | --- | --- | --- | --- | --- | --- | --- | --- | --- | --- | --- |
|  | Non-HTN (n=6,019) | HTN (n=3,206) | *P* | Non-HTN (n=12,373) | HTN (n=3,656) | *P* | Non-HTN (n=1,353) | HTN (n=1,161) | *P* | Non-HTN (n=6,052) | HTN (n=4,428) | *P* | Non-HTN (n=587) | HTN (n=372) | *P* |
| **Age**, years | 54.0±11.9 | 60.0±9.89 | <0.001 | 54.7±11.7 | 61.0±9.55 | <0.001 | 54.7±12.5 | 62.4±9.48 | <0.001 | 49.1±13.6 | 59.6±10.8 | <0.001 | 54.7±11.4 | 60.2±9.17 | <0.001 |
| **3-year average PM_1_**, µg/m^3^ | 61.1±1.70 | 61.1±1.69 | 0.650 | 55.5±1.08 | 55.4±1.07 | 0.011 | 58.1±0.88 | 58.0±0.998 | 0.342 | 57.7±1.09 | 57.6±1.02 | 0.012 | 51.6±0.19 | 51.6±0.07 | 0.349 |
| **SBP**, mmHg | 116±11.8 | 147±16.7 | <0.001 | 114±12.0 | 144±16.8 | <0.001 | 120±11.3 | 153±16.5 | <0.001 | 118±11.4 | 147±16.7 | <0.001 | 119±11.1 | 147± 15.0 | <0.001 |
| **DBP**, mmHg | 73.2±7.89 | 88.6±10.5 | <0.001 | 70.7±8.19 | 85.6±10.8 | <0.001 | 74.8±7.24 | 89.5±10.5 | <0.001 | 75.1±7.56 | 89.7±10.3 | <0.001 | 74.9±7.61 | 88.8±9.87 | <0.001 |
| **MAP**, mmHg | 87.6±8.39 | 108±11.0 | <0.001 | 85.1±8.66 | 105±11.2 | <0.001 | 89.8±7.75 | 111±10.7 | <0.001 | 89.4±8.16 | 109±10.9 | <0.001 | 89.5±8.06 | 108±9.95 | <0.001 |
| **PP**, mmHg | 43.2±8.82 | 58.0±14.4 | <0.001 | 43.2±8.84 | 58.4±14.4 | <0.001 | 45.2±8.95 | 64.0±14.9 | <0.001 | 42.9±8.14 | 57.7±14.3 | <0.001 | 43.9±8.17 | 58.6±13.6 | <0.001 |
| **BMI**，kg/m^2^ | 24.6±3.34 | 26.1±3.71 | <0.001 | 23.8±3.26 | 25.3±3.46 | <0.001 | 24.7±3.35 | 26.2±3.75 | <0.001 | 24.7±3.48 | 26.5±3.65 | <0.001 | 24.9±3.20 | 25.9±3.51 | <0.001 |
| **Sex** | | | | | | | | | | | | | | |  |
| Male | 2062 (34.3) | 1180 (36.8) | 0.015 | 5256 (42.5) | 1390 (38.0) | <0.001 | 538 (39.8) | 478 (41.2) | 0.473 | 2297 (38.0) | 1963 (44.3) | <0.001 | 195 (33.2) | 111 (29.8) | 0.274 |
| Female | 3957 (65.7) | 2026 (63.2) |  | 7117 (57.5) | 2266 (62.0) |  | 815 (60.2) | 683 (58.8) |  | 3755 (62.0) | 2465 (55.7) |  | 392 (66.8) | 261 (70.2) |  |
| **Educational level** | | | | | | | | | | | | | | |  |
| Low | 2512 (41.7) | 1764 (55.0) | <0.001 | 5809 (46.9) | 2082 (56.9) | <0.001 | 686 (50.7) | 756 (65.1) | <0.001 | 1520 (25.1) | 2053 (46.4) | <0.001 | 195 (33.2) | 171 (46.0) | <0.001 |
| Medium | 2790 (46.4) | 1168 (36.4) |  | 5156 (41.7) | 1244 (34.0) |  | 482 (35.6) | 285 (24.5) |  | 2499 (41.3) | 1562 (35.3) |  | 283 (48.2) | 154 (41.4) |  |
| High | 717 (11.9) | 274 (8.5) |  | 1408 (11.4) | 330 (9.0) |  | 185 (13.7) | 120 (10.3) |  | 2033 (33.6) | 813 (18.4) |  | 109 (18.6) | 47 (12.6) |  |
| **Marital status** | | | | | | | | | | | | | | |  |
| Married/ cohabiting | 5429 (90.2) | 2736 (85.3) | <0.001 | 11233 (90.8) | 3165 (86.6) | <0.001 | 1245 (92.0) | 1001 (86.2) | <0.001 | 5587 (92.3) | 3946 (89.1) | <0.001 | 529 (90.1) | 325 (87.4) | 0.183 |
| Widowed/single/divorced/separation | 590 (9.8) | 470 (14.7) |  | 1140 (9.2) | 491 (13.4) |  | 108 (8.0) | 160 (13.8) |  | 465 (7.7) | 482 (10.9) |  | 58 (9.9) | 47 (12.6) |  |
| **Individual income per month** | | | | | | | | | | | | | | |  |
| ≤500 RMB | 1948 (32.4) | 1215 (37.9) | <0.001 | 4565 (36.9) | 1509 (41.3) | <0.001 | 429 (31.7) | 441 (38.0) | <0.001 | 1784 (29.5) | 1772 (40.0) | <0.001 | 198 (33.7) | 136 (36.6) | 0.632 |
| 500~1000 RMB | 2298 (38.2) | 1155 (36.0) |  | 3695 (29.9) | 1058 (28.9) |  | 402 (29.7) | 365 (31.4) |  | 2078 (34.3) | 1522 (34.4) |  | 194 (33.0) | 121 (32.5) |  |
| ≥1000 RMB | 1773 (29.5) | 836 (26.1) |  | 4113 (33.2) | 1089 (29.8) |  | 522 (38.6) | 355 (30.6) |  | 2190 (36.2) | 1134 (25.6) |  | 195 (33.2) | 115 (30.9) |  |
| **Smoking** | | | | | | | | | | | | | | |  |
| Never | 4566 (75.9) | 2398 (74.8) | 0.259 | 8705 (70.4) | 2743 (75.0) | <0.001 | 969 (71.6) | 836 (72.0) | 0.829 | 4452 (73.6) | 3129 (70.7) | 0.001 | 453 (77.2) | 289 (77.7) | 0.852 |
| Ever | 1453 (24.1) | 808 (25.2) |  | 3668 (29.6) | 913 (25.0) |  | 384 (28.4) | 325 (28.0) |  | 1600 (26.4) | 1299 (29.3) |  | 134 (22.8) | 83 (22.3) |  |
| **Drinking** | | | | | | | | | | | | | | |  |
| Never | 4601 (76.4) | 2438 (76.0) | 0.670 | 9622 (77.8) | 2923 (80.0) | 0.005 | 990 (73.2) | 845 (72.8) | 0.827 | 4779 (79.0) | 3278 (74.0) | <0.001 | 504 (85.9) | 328 (88.2) | 0.303 |
| Ever | 1418 (23.6) | 768 (24.0) |  | 2751 (22.2) | 733 (20.0) |  | 363 (26.8) | 316 (27.2) |  | 1273 (21.0) | 1150 (26.0) |  | 83 (14.1) | 44 (11.8) |  |
| **High fat diet** | | | | | | | | | | | | | | |  |
| No | 4623 (76.8) | 2572 (80.2) | <0.001 | 9451 (76.4) | 3004 (82.2) | <0.001 | 1178 (87.1) | 1039 (89.5) | 0.06 | 5178 (85.6) | 3869 (87.4) | 0.007 | 493 (84.0) | 327 (87.9) | 0.093 |
| Yes | 1396 (23.2) | 634 (19.8) |  | 2922 (23.6) | 652 (17.8) |  | 175 (12.9) | 122 (10.5) |  | 874 (14.4) | 559 (12.6) |  | 94 (16.0) | 45 (12.1) |  |
| **More vegetables and fruits intake** | | | | | | | | | | | | | | |  |
| No | 4629 (76.9) | 2579 (80.4) | <0.001 | 4406 (35.6) | 1349 (36.9) | 0.151 | 1080 (79.8) | 972 (83.7) | 0.012 | 4162 (68.8) | 3076 (69.5) | 0.446 | 358 (61.0) | 224 (60.2) | 0.811 |
| Yes | 1390 (23.1) | 627 (19.6) |  | 7966 (64.4) | 2306 (63.1) |  | 273 (20.2) | 189 (16.3) |  | 1890 (31.2) | 1352 (30.5) |  | 229 (39.0) | 148 (39.8) |  |
| **Physical activity** | | | | | | | | | | | | | | |  |
| Low | 1864 (31.0) | 1266 (39.5) | <0.001 | 3060 (24.7) | 1063 (29.1) | <0.001 | 166 (12.3) | 217 (18.7) | <0.001 | 2594 (42.9) | 2152 (48.6) | <0.001 | 172 (29.3) | 134 (36.0) | 0.093 |
| Moderate | 2528 (42.0) | 1238 (38.6) |  | 5692 (46.0) | 1698 (46.4) |  | 259 (19.1) | 226 (19.5) |  | 1664 (27.5) | 1069 (24.1) |  | 266 (45.3) | 154 (41.4) |  |
| High | 1627 (27.0) | 702 (21.9) |  | 3621 (29.3) | 895 (24.5) |  | 928 (68.6) | 718 (61.8) |  | 1794 (29.6) | 1207 (27.3) |  | 149 (25.4) | 84 (22.6) |  |
| **Family history of hypertension** | | | | | | | | | | | | | | |  |
| No | 4957 (82.4) | 2318 (72.3) | <0.001 | 10589 (85.6) | 2720 (74.4) | <0.001 | 1138 (84.1) | 966 (83.2) | 0.54 | 5184 (85.7) | 3024 (68.3) | <0.001 | 461 (78.5) | 263 (70.7) | 0.006 |
| Yes | 1062 (17.6) | 888 (27.7) |  | 1784 (14.4) | 936 (25.6) |  | 215 (15.9) | 195 (16.8) |  | 868 (14.3) | 1404 (31.7) |  | 126 (21.5) | 109 (29.3) |  |
| **Type 2 diabetes** | | | | | | | | | | | | | | |  |
| No | 5453 (90.6) | 2700 (84.2) | <0.001 | 11734 (94.8) | 3247 (88.8) | <0.001 | 1228 (90.8) | 991 (85.4) | <0.001 | 5582 (92.2) | 3655 (82.5) | <0.001 | 540 (92.0) | 314 (84.4) | <0.001 |
| Yes | 562 (9.3) | 505 (15.8) |  | 632 (5.1) | 404 (11.1) |  | 120 (8.9) | 169 (14.6) |  | 451 (7.5) | 757 (17.1) |  | 43 (7.3) | 57 (15.3) |  |

Abbreviations: HTN, hypertension; PM_1_, particle matter with aerodynamic diameter ≤ 1.0μm; SBP, systolic blood pressure; DBP, diastolic blood pressure; MAP, mean arterial pressure; PP, pulse pressure; BMI, body mass index.

Notes: Data are the mean ± standard deviation for continuous variables and number (percentage) for categorical variables.

**Table S2 Odds ratios and 95% confidence intervals of hypertension associated with an increment of 1 µg/m3 in PM_1_ concentration, stratified by potential modifiers**

| Modifiers | Odd ratio | 95% confidence intervals | | *P* _interaction_ |
| --- | --- | --- | --- | --- |
| Gender |  |  |  |  |
| Male | 1.079 | 1.063, 1.094 | | < 0.001 |
| Female | 1.021 | 1.009, 1.033 | |  |
| Age |  |  |  |  |
| <65 | 1.036 | 1.025, 1.047 | | 0.705 |
| ≥65 | 1.039 | 1.023, 1.056 | |  |
| Smoking |  |  |  |  |
| Never | 1.028 | 1.017, 1.039 | | < 0.001 |
| Ever | 1.085 | 1.067, 1.104 | |  |
| Drinking |  |  |  |  |
| Never | 1.032 | 1.021, 1.043 | | < 0.001 |
| Ever | 1.080 | 1.060, 1.100 | |  |
| More vegetables and fruits intake | | |  |  |
| Yes | 1.066 | 1.049, 1.083 | | 0.001 |
| No | 1.032 | 1.020, 1.043 | |  |
| High fat diet | |  |  |  |
| No | 1.034 | 1.024, 1.045 | | < 0.001 |
| Yes | 1.078 | 1.057, 1.099 | |  |
| Physical activity | |  | |  |
| Low | 1.040 | 1.024, 1.056 | | Reference |
| Moderate | 1.033 | 1.019, 1.048 | | 0.522 |
| High | 1.063 | 1.044, 1.081 | | 0.067 |

Legend: Adjusted for sex, age, marital status, education level, income smoking, alcohol drinking, physical activity, high fat diet, vegetables and fruits intake, family history of hypertension, body mass index, type 2 diabetes.

**Table S3** Changes in blood pressure (mmHg) associated with an increment of 1µg/m^3^ in long-term PM_1_ concentration, stratified by potential modifiers

| Modifiers | Systolic blood pressure ^a^ | | Diastolic blood pressure ^a^ | | Mean Arterial Pressure ^a^ | | Pulse Pressure ^a^ | |
| --- | --- | --- | --- | --- | --- | --- | --- | --- |
|  | mmHg (95%CI) | P_interaction_ | mmHg (95%CI) | P_interaction_ | mmHg (95%CI) | P_interaction_ | mmHg (95%CI) | P_interaction_ |
| Gender |  |  |  |  |  |  |  |  |
| Male | 0.680 (0.577, 0.784) | < 0.001 | 0.512 (0.449, 0.575) | < 0.001 | 0.568 (0.496, 0.640) | < 0.001 | 0.169 (0.101, 0.236) | < 0.001 |
| Female | 0.230 (0.148, 0.312) |  | 0.215 (0.165, 0.266) |  | 0.220 (0.163, 0.278) |  | 0.014 (-0.039, 0.068) |  |
| Age |  |  |  |  |  |  |  |  |
| <65 | 0.352 (0.275, 0.429) | 0.682 | 0.322 (0.276, 0.368) | 0.938 | 0.332 (0.279, 0.385) | 0.878 | 0.030 (-0.020, 0.081) | 0.486 |
| ≥65 | 0.383 (0.252, 0.514) |  | 0.318 (0.240, 0.396) |  | 0.340 (0.250, 0.430) |  | 0.065 (-0.021, 0.151) |  |
| Smoking |  |  |  |  |  |  |  |  |
| Never | 0.307 (0.231, 0.383) | < 0.001 | 0.268 (0.221, 0.314) | < 0.001 | 0.281 (0.228, 0.334) | < 0.001 | 0.039 (-0.010, 0.088) | 0.006 |
| Ever | 0.664 (0.540, 0.787) |  | 0.497 (0.421, 0.572) |  | 0.552 (0.466, 0.638) |  | 0.167 (0.087, 0.247) |  |
| Drinking |  |  |  |  |  |  |  |  |
| Never | 0.321 (0.246, 0.395) | < 0.001 | 0.274 (0.229, 0.320) | < 0.001 | 0.290 (0.238, 0.342) | < 0.001 | 0.046 (-0.002,0.094) | 0.018 |
| Ever | 0.671 (0.539, 0.803) |  | 0.509 (0.428, 0.589) |  | 0.563 (0.470, 0.655) |  | 0.163 (0.077, 0.248) |  |
| More vegetables and fruits intake | |  |  |  |  |  |  |  |
| Yes | 0.631 (0.520, 0.741) | < 0.001 | 0.505 (0.437, 0.573) | < 0.001 | 0.547 (0.470, 0.624) | < 0.001 | 0.126 (0.054, 0.197) | 0.074 |
| No | 0.278 (0.196, 0.359) |  | 0.233 (0.183, 0.283) |  | 0.248 (0.191, 0.305) |  | 0.045 (-0.008, 0.098) |  |
| High fat diet | |  |  |  |  |  |  |  |
| No | 0.359 (0.285, 0.432) | 0.012 | 0.300 (0.255, 0.345) | 0.006 | 0.319 (0.268, 0.371) | 0.005 | 0.059 (0.011, 0.107) | 0.205 |
| Yes | 0.555 (0.419, 0.692) |  | 0.432 (0.348, 0.516) |  | 0.473 (0.377, 0.569) |  | 0.123 (0.035, 0.212) |  |
| Physical activity | |  |  |  |  |  |  |  |
| Low | 0.314 (0.200, 0.427) | Reference | 0.254 (0.184, 0.323) | Reference | 0.274 (0.194, 0.353) | Reference | 0.060 (-0.014, 0.134) | Reference |
| Moderate | 0.246 (0.148, 0.344) | 0.370 | 0.270 (0.210, 0.330) | 0.726 | 0.262 (0.193, 0.331) | 0.824 | -0.024 (-0.087,0.040) | 0.087 |
| High | 0.761 (0.636, 0.885) | < 0.001 | 0.514 (0.438, 0.590) | < 0.001 | 0.596 (0.509, 0.684) | < 0.001 | 0.247 (0.166, 0.328) | < 0.001 |

Abbreviations: PM_1_, particle matter with aerodynamic diameter ≤ 1.0μm; CI, confidence interval.

^a^ Adjusted for sex, age, marital status, education level, income smoking, alcohol drinking, physical activity, high fat diet, vegetables and fruits intake, family history of hypertension, body mass index, type 2 diabetes, hypertension medicine use

**Table S4 Effects of long-term exposure to PM_1_ on Hypertension and Blood pressure in sensitivity analysis**

| Types of sensitivity analysis | OR of hypertension (95% CI) | Changes in mmHg (95% CI) | | | |
| --- | --- | --- | --- | --- | --- |
|  |  | SBP | DBP | MAP | PP |
| Using different average concentration of PM_1_ | |  | | | |
| 1-year average | 1.026 (1.018, 1.035) | 0.284 (0.225, 0.343) | 0.246 (0.210, 0.282) | 0.259(0.218, 0.300) | 0.038 (0.000, 0.076) |
| 2-year average | 1.034 (1.025, 1.043) | 0.311 (0.251, 0.372) | 0.273 (0.236, 0.310) | 0.286(0.243, 0.328) | 0.039 (0.000, 0.078) |
| 4-year average | 1.051 (1.042, 1.061) | 0.480 (0.415, 0.546) | 0.375 (0.335, 0.415) | 0.410(0.364, 0.456) | 0.106 (0.063, 0.148) |
| 5-year average | 1.047 (1.036, 1.057) | 0.448 (0.378, 0.519) | 0.358 (0.314, 0.401) | 0.388 (0.338, 0.437) | 0.091 (0.045, 0.137) |
| Adjusted for survey site | 1.017 (0.997, 1.037) | 0.169 (0.035, 0.303) | 0.208 (0.126, 0.290) | 0.195 (0.102, 0.289) | −0.039 (−0.127, 0.049) |

Abbreviations: PM_1_, particle matter with aerodynamic diameter ≤ 1.0μm; OR, odds ratio; CI, confidence interval; SBP, systolic blood pressure; DBP, diastolic blood pressure; MAP, mean arterial pressure; PP, pulse pressure
